# Influence of Europium Doping on the Electrical Properties of Ba_0.9_Ca_0.1_TiO_3_ Ceramics

**DOI:** 10.3390/ma19204394

**Published:** 2026-10-19

**Authors:** Dawid Kurczek, Jolanta Makowska, Tomasz Pikula, Rafał Panek, Grzegorz Tytko, Małgorzata Karolus, Małgorzata Adamczyk-Habrajska

**Affiliations:** 1 Institute of Materials Engineering, Faculty of Science and Technology, University of Silesia, 75 Pułku Piechoty 1A, 41-500 Chorzów, Poland; dawid.kurczek@us.edu.pl (D.K.); malgorzata.karolus@us.edu.pl (M.K.); malgorzata.adamczyk-habrajska@us.edu.pl (M.A.-H.); 2 Department of Electronics and Information Technology, Faculty of Electrical Engineering and Computer Science, Lublin University of Technology, 20-618 Lublin, Poland; t.pikula@pollub.pl; 3 Department of Construction Materials Engineering and Geoengineering, Faculty of Civil Engineering and Architecture, Lublin University of Technology, 40 Nadbystrzycka Str., 20-618 Lublin, Poland; r.panek@pollub.pl; 4 Department of Digital Systems, Faculty of Automatic Control, Electronics and Computer Science, The Silesian University of Technology, Akademicka 16, 44-100 Gliwice, Poland; grzegorz.tytko@polsl.pl

**Keywords:** Ba_0.9_Ca_0.1_TiO_3_, europium doping, impedance spectroscopy, dielectric properties, perovskite ceramics

## Abstract

Eu-doped Ba_0.9_Ca_0.1_TiO_3_ (BCT10) ceramics with the nominal composition (Ba_0.9−x_Eu_x_Ca_0.1_)TiO_3_, where x = 0–0.025, were prepared by the conventional solid-state reaction method. X-ray diffraction analysis and Rietveld refinement revealed a predominantly tetragonal perovskite structure (space group P4mm), with a gradual reduction in tetragonal distortion as the Eu content increased. Scanning electron microscopy showed the grains shrinking steadily with dopant content, from 15.2 µm in the undoped ceramic to about 1.4 µm at the highest doping level. EDS confirmed the presence of the constituent elements and indicated a relatively uniform elemental distribution within the examined region. Impedance spectroscopy revealed thermally activated conduction with two relaxation processes attributed to grains and grain boundaries, described by an equivalent circuit consisting of two Voigt elements incorporating constant phase elements. The activation energies of the first processes decreased with Eu addition, reaching their lowest values at x = 0.01. The subsequent differences in their concentration dependence suggest that Eu doping modifies the electrical transport processes in grains and grain boundaries. These changes, together with the pronounced grain refinement, may be associated with modifications in defect chemistry and grain-boundary contributions to the overall electrical response. Overall, Eu^3+^ doping provides an effective means of modifying the microstructure and electrical properties of BCT10 ceramics.

## 1. Introduction

Barium titanate BaTiO_3_ has long been recognized as one of the most important lead-free ferroelectric materials with a perovskite structure [1,2,3,4]. Its high dielectric permittivity, good piezoelectric response, and well-established sequence of phase transitions make it attractive for a wide range of functional applications. Moreover, its relatively simple chemical composition and the absence of toxic lead make BaTiO_3_ a promising alternative to Pb(Zr,Ti)O_3_ (PZT)-based materials, which are still widely used in electromechanical devices [5]. Growing restrictions on lead-containing materials have increased interest in lead-free alternatives with properties comparable to those of PZT [6,7,8].

One of the most effective approaches to tailoring the properties of BaTiO_3_ is compositional modification through ionic substitution at the A and B sites of the perovskite lattice [9]. In this context, the (Ba,Ca) (Zr,Ti)O_3_ system has attracted considerable attention. In this system, Ba^2+^ is partially replaced by Ca^2+^ at the A site, while Ti^4+^ is partially substituted by Zr^4+^ at the B site [10,11]. An appropriate choice of composition promotes the coexistence of ferroelectric phases with an enhanced electromechanical response, often associated with a morphotropic phase boundary (MPB), resulting in a significant increase in the piezoelectric coefficient d_33_ [12,13,14,15,16]. Consequently, ceramics from this system can exhibit piezoelectric properties competitive with those of some lead-containing materials [17].

Ba_1−x_Ca_x_TiO_3_ (BCT) solid solutions represent a simpler compositional modification, in which Ba^2+^ is partially substituted by Ca^2+^ while the B site remains occupied by Ti^4+^ [3]. The incorporation of Ca affects phase stability, phase-transition temperatures, and the dielectric and ferroelectric behavior of the material [18,19,20]. Thus, relatively small changes in Ca content provide an effective means of tailoring the functional response of BCT, making this system interesting from both fundamental and application-oriented perspectives [21,22].

Among the Ba_1−x_Ca_x_TiO_3_ compositions, Ba_0.9_Ca_0.1_TiO_3_ (BCT10) has attracted particular interest. Previous studies have examined the relationship between processing conditions, structure, microstructure, and functional properties of this composition. BCT10 ceramics prepared by the Pechini method have been reported to exhibit favorable piezoelectric properties [23], while microwave-assisted hydrothermal synthesis has been explored as an alternative preparation route [24]. The effects of processing conditions on the phase structure and microstructure have also been investigated [25]. In addition, the pyroelectric response of BCT10 suggests its potential for energy-conversion applications [26].

In addition to isovalent substitution, heterovalent doping provides another effective route for modifying the properties of BaTiO_3_-based ceramics. Rare-earth and transition-metal ions are particularly interesting in this respect because their incorporation can alter the defect chemistry of the perovskite lattice [27,28]. The resulting effect depends primarily on the ionic radius and valence state of the dopant, as well as on its preferred lattice site. Ions with radii comparable to that of Ti^4+^ may occupy the B site and act as acceptor dopants, whereas larger ions with higher valence states may substitute for Ba^2+^ or Ca^2+^ at the A site and exhibit donor behavior. Such heterovalent substitutions require charge compensation, leading to changes in the type and concentration of point defects and, consequently, in the electrical behavior of the material [29]. The effects of doping in BaTiO_3_ strongly depend on the nature of the incorporated ion. Manganese doping, for example, has been shown to modify dielectric and ferroelectric properties, grain size, and domain-switching behavior [30,31]. Nickel can affect phase-transition temperatures and the stability ranges of individual crystallographic phases [31,32], whereas Cr^3+^ has been reported to alter the local structure and ferroelectric response of BaTiO_3_ [33,34,35]. These findings demonstrate that even relatively small dopant concentrations can significantly modify the defect structure, microstructure, and functional behavior of BaTiO_3_-based ceramics. Among the various dopants, rare-earth ions are of particular interest because their ionic radii and valence states may allow them to occupy different lattice sites within the perovskite structure [36,37,38]. Europium Eu^3+^ is especially interesting in this regard. Depending on the host composition, dopant concentration, and processing conditions, Eu^3+^ may exhibit different site preferences in BaTiO_3_-based materials [36,37,38,39]. Its lattice position determines the associated charge-compensation mechanism and therefore influences the type and concentration of defects formed in the ceramic [40].

Previous studies of Eu^3+^-doped (Ba,Ca)TiO_3_ have focused mainly on optical phenomena, particularly photoluminescence and photochromic behavior [41,42]. In contrast, considerably less attention has been paid to the effect of Eu^3+^ on the dielectric and electrical properties of this system. Studies of other heterovalent dopants in BaTiO_3_ have demonstrated that changes in defect chemistry can strongly affect dielectric permittivity, dielectric losses, and electrical conductivity [35,43,44]. Donor dopants such as Sb, Nb, and Ta have also been shown to modify the temperature dependence of the dielectric response [36]. The associated charge-compensation mechanisms and point defects have been investigated using defect-sensitive techniques, including positron annihilation spectroscopy and electron paramagnetic resonance [45,46].

The lattice site occupied by Eu^3+^ may therefore be particularly important for the electrical behavior of Ba_0.9_Ca_0.1_TiO_3_. Substitution of Ba^2+^ or Ca^2+^ by Eu^3+^ corresponds to donor-type doping and requires compensation of the excess positive charge.

Literature studies indicate that in barium titanate-based solid solutions, europium may occur as the metastable Eu^2+^ ion with a partially filled 4f shell, or as a mixture of Eu^2+^ (4f^7^) and Eu^3+^ (4f^6^) ions [47]. The ionic radius of Eu^3+^ is 1.12 Å (for coordination number 9). It is thus smaller than the ionic radii of Ba^2+^ and Ca^2+^ and, at the same time, larger than that of Ti^4+^ (Table 1).

Based on the work of M.T. Buscaglia et al. [49] on the substitution of Pr^3+^ and La^3+^ ions, which are of similar size, it was assumed that in Ba_0.9_Ca_0.1_TiO_3_ ceramics, Eu^3+^ ions enter the A sublattice of the perovskite cell in place of Ba^2+^ (or Ca^2+^) ions, as implied by the nominal composition (Ba_0.9−x_Eu_x_Ca_0.1_)TiO_3_. When synthesis is carried out in an oxidizing atmosphere (air), the incorporation of Eu^3+^ into the A sublattice in place of the divalent Ba^2+^ (or Ca^2+^) cation leads to the formation of a positively charged donor defect, Eu^•^_Ba_ [50]. To maintain the electroneutrality of the material, the resulting excess positive charge must be compensated by the formation of defects with a negative effective charge. This can occur in three different ways.

1. Compensation by A-site cation vacancies. The incorporation of two Eu^3+^ ions creates one A-site cation vacancy (V″_Ba_) with a double negative effective charge, as described by Equation (1):

(1) $${\mathrm{Eu}}_{2}O_{3}+3\;\mathrm{Ti}O_{2\;}\;\to 2\;{\mathrm{Eu}}_{\mathrm{Ba}}^{•}+V_{\mathrm{Ba}}^{\prime \prime }+3\;{\mathrm{Ti}}_{\mathrm{Ti}}^{\times }+9\;O_{O}^{\times }\;$$

This mechanism is characterized by a lower formation energy than vacancy formation in the B sublattice [51].

2. Compensation by B-site cation vacancies. For every four Eu^•^_Ba_ donor ions incorporated, one titanium vacancy (V⁗_Ti_) with a fourfold negative effective charge is formed (Equation (2)):

(2) $${2\;\mathrm{Eu}}_{2}O_{3}+3\;\mathrm{Ti}O_{2\;}\;\to 4\;{\mathrm{Eu}}_{\mathrm{Ba}}^{•}+V_{\mathrm{Ti}}^{⁗}+3\;{\mathrm{Ti}}_{\mathrm{Ti}}^{\times }+12\;O_{O}^{\times }$$

Theoretical calculations (e.g., lattice statics methods) indicate that the formation energy of V⁗_Ti_ is higher than that of V″_Ba_ [51]. Nevertheless, this mechanism may occur locally, particularly under oxygen-rich conditions or in the case of slight deviations from B-site stoichiometry in the starting mixture of reagents [52].

3. Electronic compensation through the reduction of Ti^4+^ to Ti^3+^ (free electrons) (Equation (3)).

(3) Eu2O3+2 TiO2  →2 EuBa•+2 TiTi×+6 OO×+12O2g+2 e′

In this mechanism, the incorporation of Eu^3+^, combined with the release of oxygen from the crystal lattice, generates free charge carriers (electrons or Ti^3+^ small polarons). In synthesis in pure air, this mechanism is suppressed in favor of cation-vacancy compensation, which prevents the ceramic from becoming a highly conductive semiconductor [53,54]. It is also worth noting that the role of this mechanism increases at higher sintering temperatures and under reduced oxygen partial pressure (reducing atmosphere). It is therefore an unlikely mechanism in the case of free sintering in air [50,53].

Despite these findings, the effect of Eu^3+^ doping on the electrical properties of Ba_0.9_Ca_0.1_TiO_3_ has not yet been fully investigated. In particular, little is known about how Eu^3+^ affects the crystal structure, microstructure, and electrical transport in BCT10 ceramics. A better understanding of these effects may help clarify the role of Eu^3+^ in BCT and determine whether europium doping can be used to modify its functional properties.

Therefore, the aim of this study was to investigate the effect of europium doping on the electrical properties of Ba_0.9_Ca_0.1_TiO_3_ ceramics. The samples were characterized by scanning electron microscopy (SEM), while their electrical properties were studied using impedance spectroscopy measurements. The results were analyzed to determine the role of Eu^3+^ in the electrical behavior of BCT10 ceramics.

## 2. Materials and Methods

Ba_0.9_Ca_0.1_TiO_3_ ceramics, undoped and doped with Eu, with the nominal composition (Ba_0.9−x_Eu_x_Ca_0.1_)TiO_3_, where x = 0, 0.005, 0.010, 0.015, 0.020 and 0.025, were prepared by the conventional solid-state reaction method. Hereafter, the undoped sample is denoted as BCT, and the doped samples as BCT:Eu0.5, BCT:Eu1, BCT:Eu1.5, BCT:Eu2 and BCT:Eu2.5, where the number gives the nominal Eu content in mol% relative to the A-site sublattice (i.e., 100x); for example, BCT:Eu0.5 corresponds to x = 0.005. BaCO_3_ (Sigma-Aldrich, St. Louis, MO, USA), CaCO_3_ (Sigma-Aldrich, St. Louis, MO, USA), TiO_2_ (Avantor, Gliwice, Poland) and Eu_2_O_3_ (Sigma-Aldrich, St. Louis, MO, USA), powders of analytical purity (≥99.9%) were used as starting materials and weighed in stoichiometric proportions according to the nominal composition.

The precursor powders were wet-milled in 96% ethanol using a planetary mill. Yttria-stabilized zirconia balls were used as the milling media. Milling was performed for t = 24 h at 250 rpm to ensure homogeneous mixing and reduce the particle size of the starting powders. After milling, the ethanol was evaporated, and the dried powders were transferred to corundum crucibles. The powder mixtures were calcined in a muffle furnace at *T* = 1000 °C for *t* = 4 h using a heating rate of 5 °C/min. After calcination and natural cooling to room temperature, the powders were initially ground in a mortar and subsequently wet-milled again in ethanol for *t* = 24 h under the same conditions. The second milling step was used to break down agglomerates formed during calcination and obtain homogeneous powders suitable for further processing. The resulting powders were uniaxially pressed (*p* = 200 MPa) into cylindrical pellets using a semi-automatic hydraulic press and a steel die with a diameter of *d* = 10 mm. The pellets were placed in corundum crucibles on a high-purity Al_2_O_3_ substrate and sintered in air in a high-temperature chamber furnace. The samples were heated at a rate of 5 °C/min to 1500 °C for *t* = 3 h, and subsequently cooled naturally to room temperature. The fabricated ceramic materials were subjected to comprehensive characterization to determine the relationships between structure, microstructure, and functional properties.

The bulk density of the sintered ceramics was measured by the Archimedes method in distilled water.

The phase composition of the prepared samples was evaluated using X-ray diffraction (XRD) (PANalytical B.V., Almelo, The Netherlands) on a Panalytical X’pert PRO MPD diffractometer (Eindhoven, The Netherlands) in the standard Θ–2Θ configuration. The diffractometer was equipped with a PW 3050 goniometer, a PIXcel 1D detector, and a copper (Cu) radiation source (CuKα = 1.54178 Å). For phase and structural analysis, the HighScore Plus 3.0e software was used, incorporating the 2022 edition of the PDF-2 DL database from JCPDS-ICDD.

The surface morphology, microstructure, and grain size of the sintered ceramics were evaluated using a JEOL JSM-7100F TTL LV scanning electron microscope (SEM, JEOL Ltd., Tokyo, Japan). Energy-dispersive X-ray spectroscopy (EDS), integrated with the SEM, was used to identify the constituent elements and examine their spatial distribution in the selected ceramic sample.

Microstructural observations were performed on the fractured surfaces of the ceramics. Prior to measurement, the samples were rinsed in ethyl alcohol in an ultrasonic cleaner for one hour to remove contaminants. The sample surfaces were then sputter-coated with a thin gold layer in order to obtain high-quality images in secondary electron (SE) mode and to prevent beam drift caused by electrostatic charge accumulation.

Grain-size analysis was performed using SEM micrographs and image analysis. Individual grain dimensions were determined from the microstructural images, and the resulting measurements were statistically evaluated to calculate the mean grain size and standard deviation for each composition. Grain-size distribution histograms were constructed to assess microstructural homogeneity and examine the distribution shape, dispersion, and possible deviations from unimodal behavior [55].

The electric properties were determined using a precision LCR meter, Agilent E4980A. Samples intended for electrical measurements were prepared to a final thickness of approximately *d* = 1 mm. Silver paste was applied to both parallel surfaces to form electrodes, which were subsequently fired at *T* = 850 °C for *t* = 30 min to ensure good adhesion and electrical contact.

The impedance measurements of ceramics were performed using a computer-controlled impedance analyzer (Agilent E4980A LCR meter, Keysight Technologies, Inc., Santa Rosa, CA, USA) over a frequency range of 20 Hz to 2 MHz and a temperature range of 300–825 K. The internal consistency, quality, and physical causality of the experimental impedance data obtained by the IS method were verified using Kramers–Kronig (K-K) relations [56].

## 3. Results and Discussion

### 3.1. X-Ray Analysis

**Figure 1:**
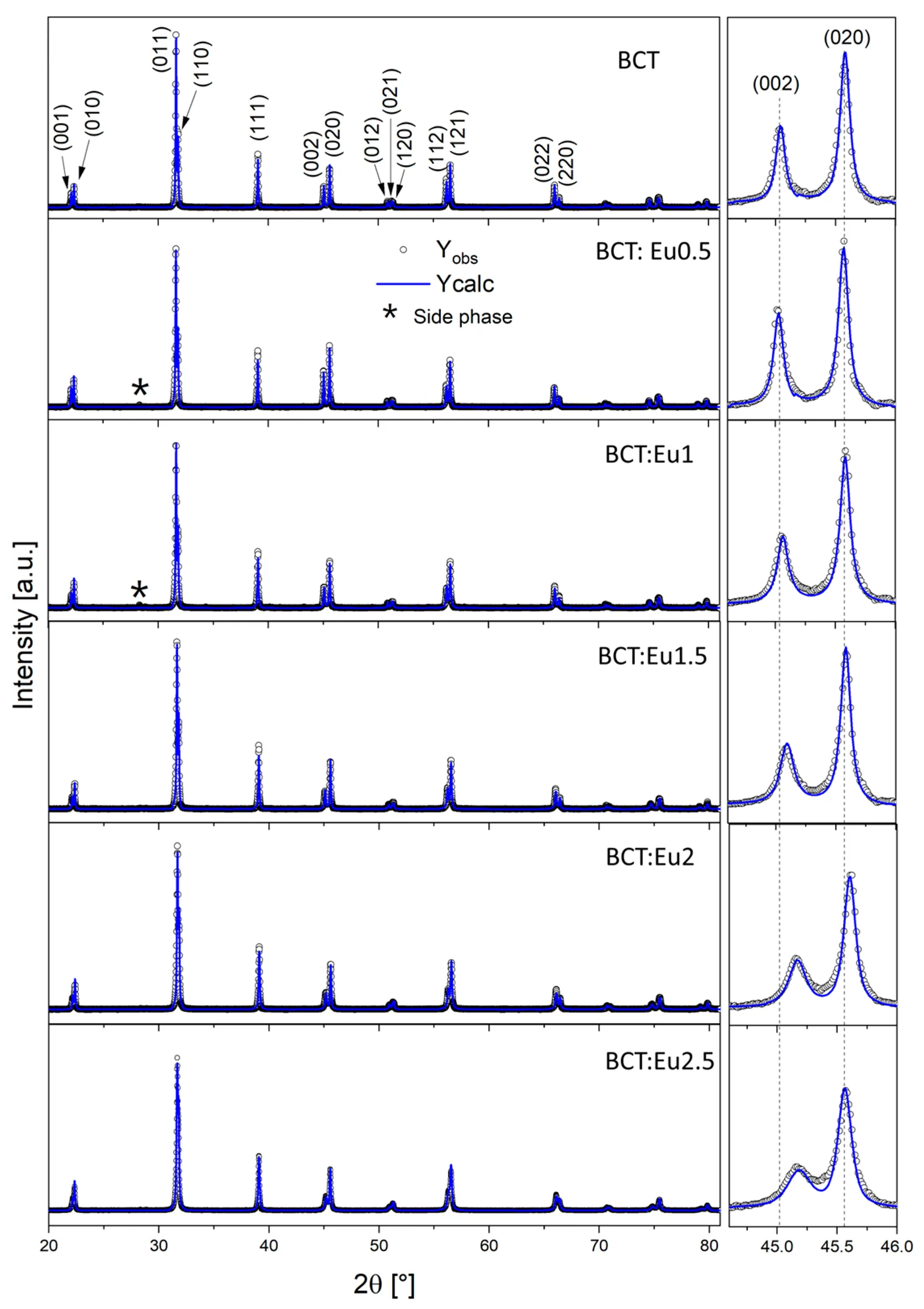
The results of XRD investigations for Eu-doped BCT samples. The right panel shows a zoom-in of the (002) and (020) doublet in the range of 44.5–46° 2θ.

X-ray diffraction analysis was performed to determine the phase composition and crystal structure of the investigated ceramics and to evaluate the effect of Eu^3+^ substitution on their structural characteristics. Figure 1 shows XRD patterns recorded for (Ba_0.9−x_Eu_x_Ca_0.1_)TiO_3_ ceramics.

**Figure 2:**
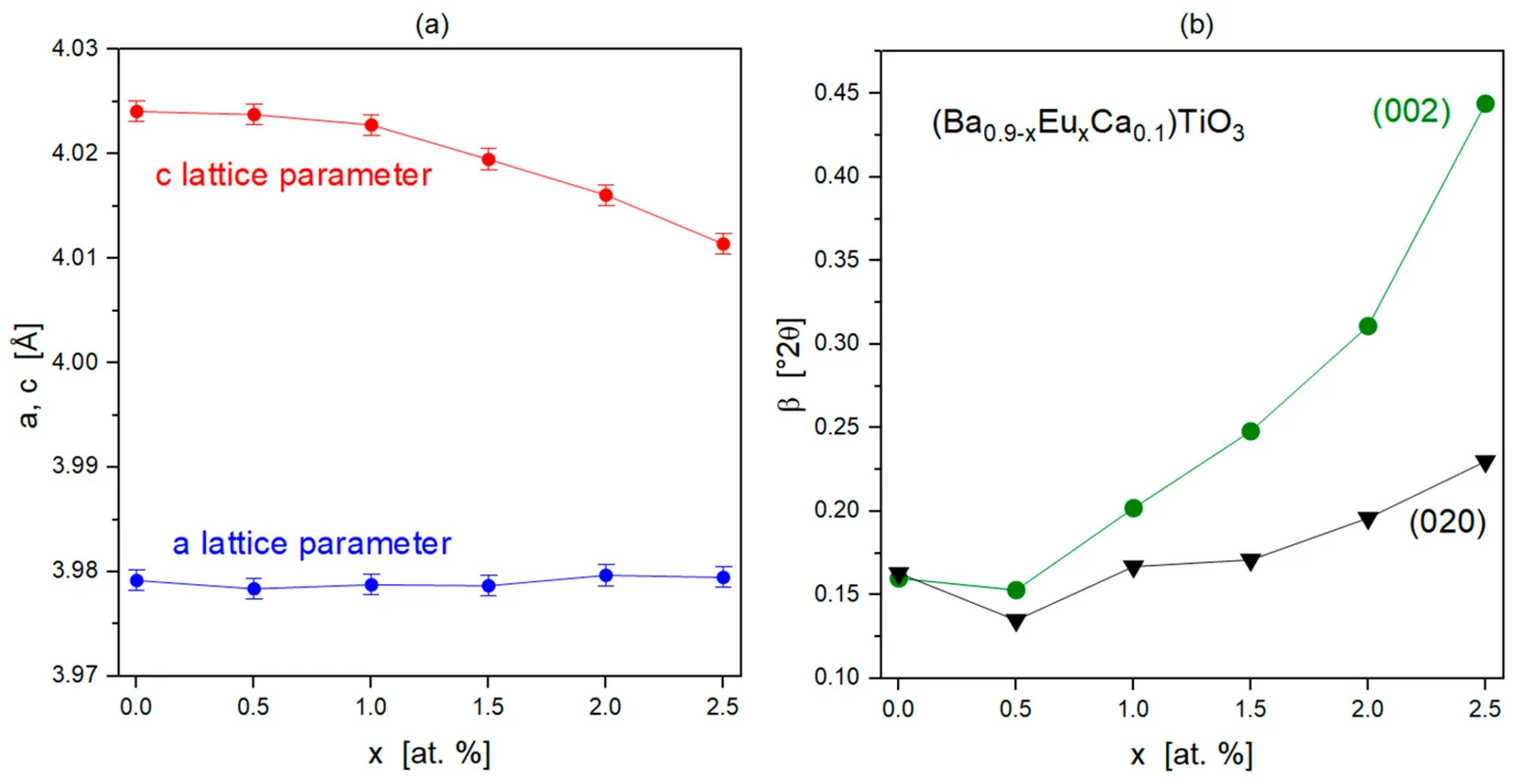
The values of lattice parameters derived from Rietveld refinement of XRD data for Eu-doped BCT samples (**a**) and the integral breadth of the (002) and (020) peaks (**b**).

All the investigated samples adopt a tetragonal structure (P4mm space group), characteristic of BaTiO_3_. Tiny amounts of secondary phase were detected in the case of BCT:Eu0.5 and BCT:Eu1. At first glance, all the diffractograms look similar, but closer inspection reveals substantial differences. The right panel of Figure 1 shows a zoom-in of the (002) and (020) doublet in the 44.5–46° 2θ range. The (020) peak does not noticeably change its position with increasing Eu concentration in the crystal lattice. The same trend was observed for all peaks in the (0k0) family. In contrast, the (002) peak and all peaks in the (00*l*) family shift to higher 2θ angles and broaden significantly as Eu concentration increases. This shows that doping BCT with Eu^3+^ ions up to 2.5 at.% causes structural changes mainly along the z crystallographic direction. To quantify these changes, Rietveld refinement was applied to all diffractograms, and the results were summarized in Figure 2 and Table 2.

Figure 2a shows how the a and c lattice parameters vary with increasing Eu concentration. As the a parameter remains constant at around 3.979 Å, the c parameter decreases gradually from 4.024 Å in the BCT sample to 4.011 Å in the BCT:Eu2.5 sample. This corresponds to a decrease in the degree of tetragonal deformation of the elemental cell from 1.13 to 0.8%. The drop in the c parameter and the decrease in unit cell volume can be explained by substituting Ba^2+^ ions with the smaller Eu^3+^ ions, as indicated by the ionic radii listed in Table 1. Figure 2b shows the evolution of the integral breadth, β, of the (002) and (020) peaks with the growth of Eu concentration. The anisotropic broadening of the diffraction peaks is clearly visible. While the (020) peak breadth increases slightly from 0.16 to 0.23, the (002) peak breadth increases almost three-fold, from 0.16° to 0.45°. This confirms that introducing Eu into the BCT crystal lattice not only reduces crystal grain size, as revealed by SEM, but also increases microstrains along the z crystallographic direction.

### 3.2. Analysis of Microstructure and Chemical Composition

**Figure 3:**
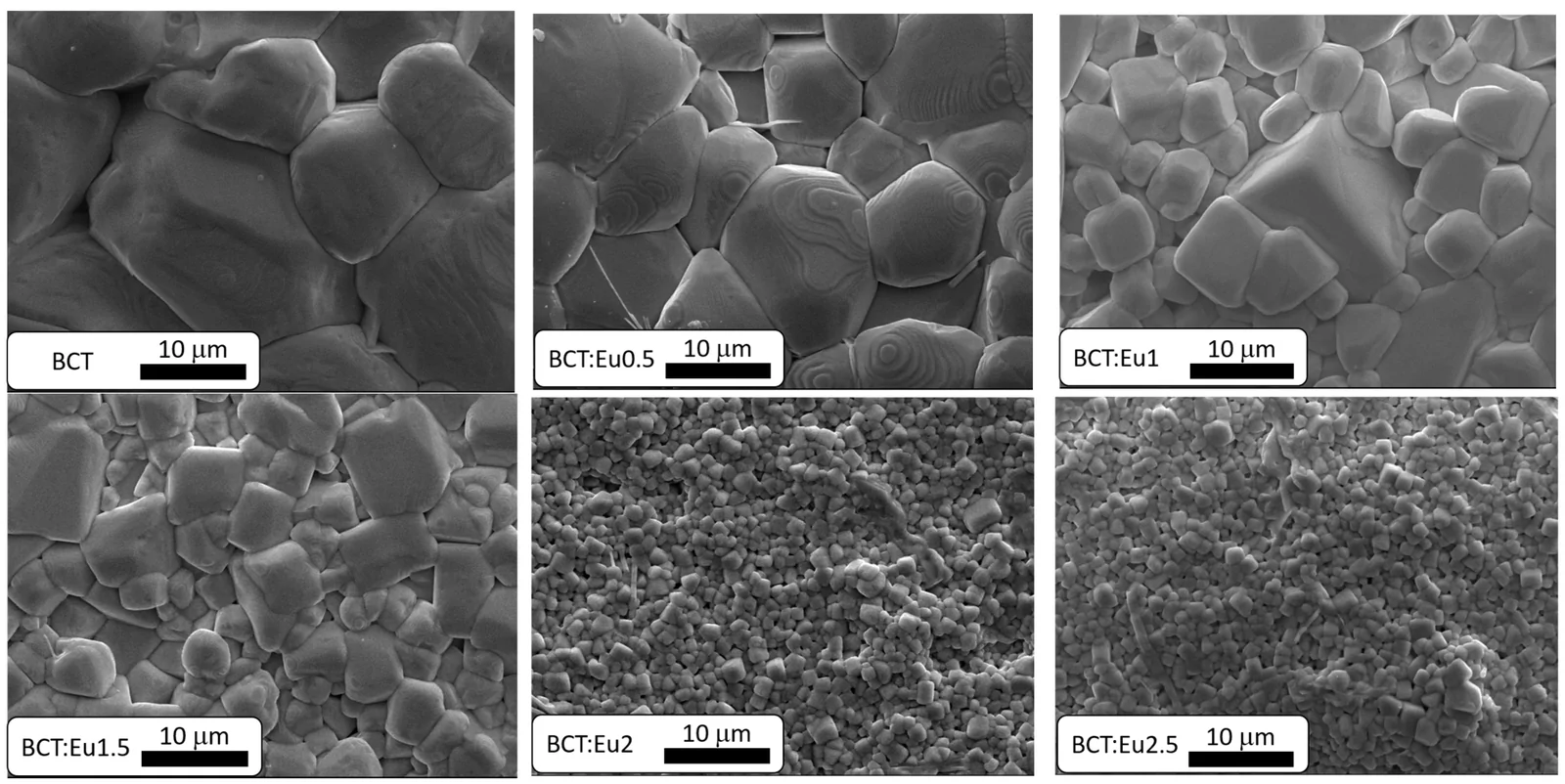
Microstructure images of (Ba_0.9−x_Eu_x_Ca_0.1_)TiO_3_ ceramics (magnification 2500).

Figure 3 shows SEM images of the microstructure of (Ba_0.9−x_Eu_x_Ca_0.1_)TiO_3_ ceramics with different Eu^3+^ concentrations.

**Figure 4:**
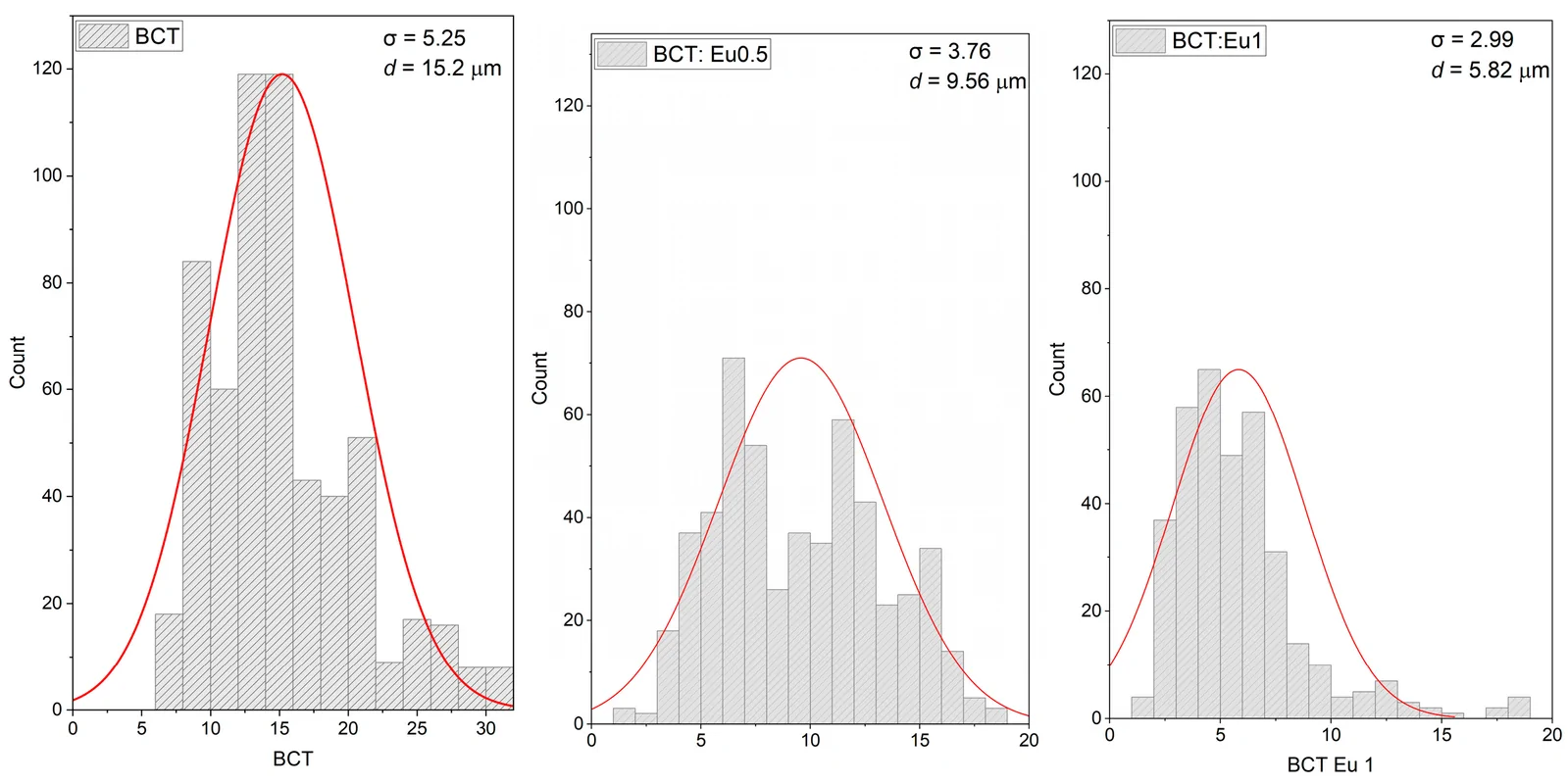

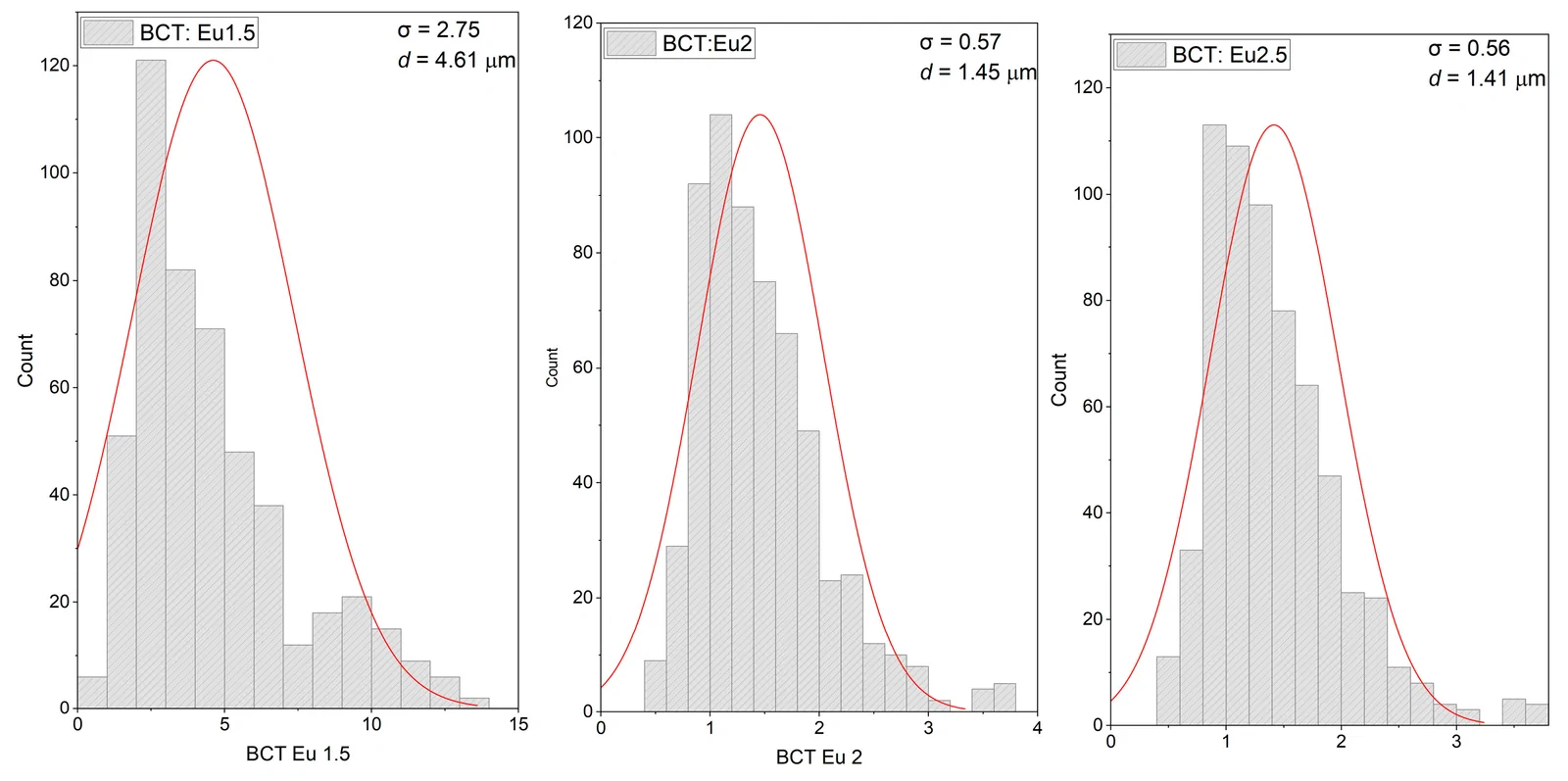
Grain size distribution of BCT:Eu^3+^ ceramics.

The undoped sample (x = 0) is characterized by relatively large, well-developed grains with irregular polygonal shapes. A similar coarse-grained microstructure is observed for the sample with the lowest Eu content (BCT:Eu0.5). With increasing Eu concentration, a clear decrease in grain size is observed. For BCT:Eu1 and BCT:Eu1.5, the microstructure becomes progressively finer, with an increasing fraction of smaller grains. The most pronounced grain refinement is observed for BCT:Eu2 and BCT:Eu2.5, where fine grains dominate the microstructure. The lower-magnification images presented in the insets confirm that these changes occur throughout larger areas of the samples. A similar reduction in grain size upon Eu^3+^ addition has previously been reported for BaTiO_3_-based ceramics [57], indicating that rare-earth doping can significantly affect microstructural development during sintering [58]. Thus, the observed evolution of the microstructure confirms that Eu^3+^ incorporation provides an effective way to modify the grain structure of BCT ceramics. The grain size distributions presented in Figure 4 confirm the changes observed in the SEM images.

The undoped BCT ceramic has the largest grains, with an average grain size of 15.2 μm. With increasing Eu^3+^ content, the grain size gradually decreases. For BCT:Eu0.5, the average grain size is 9.56 μm, while for BCT:Eu1 and BCT:Eu1.5 it decreases to 5.82 and 4.61 μm, respectively. A much stronger reduction is observed for BCT:Eu2, where the average grain size is only 1.45 μm. Increasing the Eu^3+^ content to BCT:Eu2.5 causes almost no further change, with an average grain size of 1.41 μm. The grain size distribution also becomes narrower with increasing Eu^3+^ content. The standard deviation decreases from 5.25 for the undoped BCT to 0.56–0.57 for BCT:Eu2 and BCT:Eu2.5. This shows that the addition of europium not only reduces the grain size but also makes the microstructure more uniform. These results are consistent with the SEM observations and with previous studies on Eu-doped BaTiO_3_-based ceramics [47,59].

**Figure 5:**
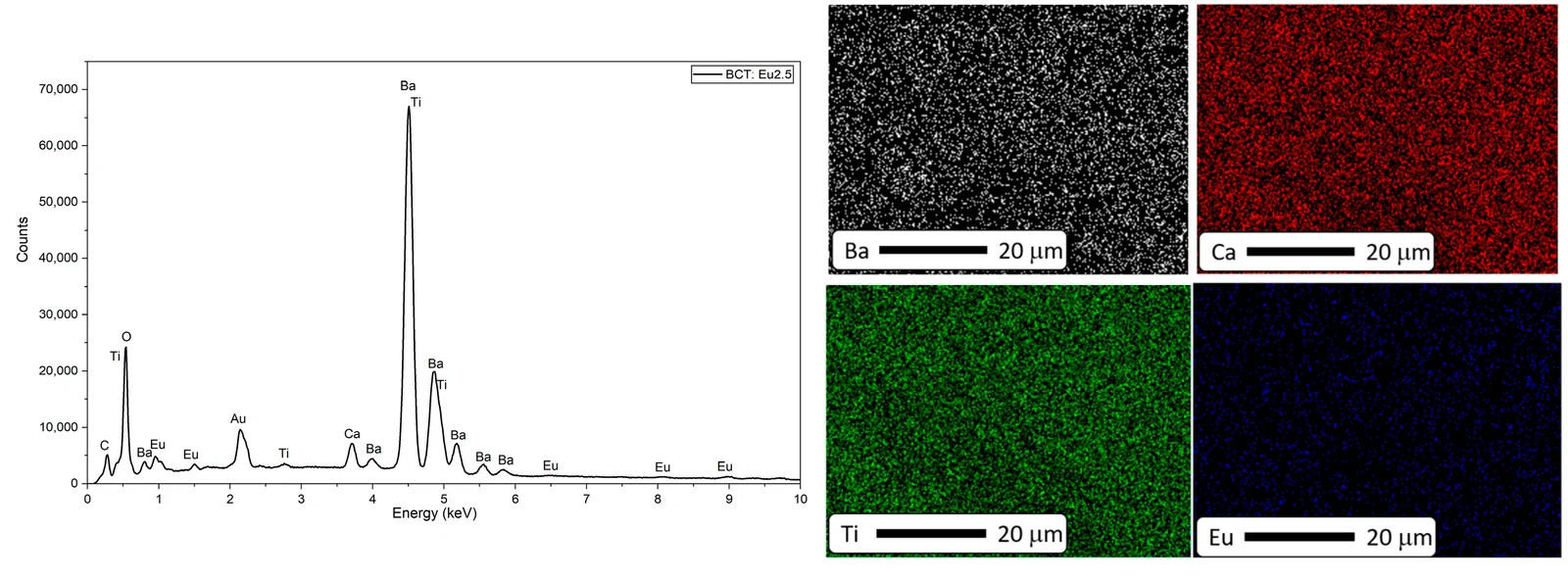
EDS spectrum and elemental distribution maps of Ba, Ca, Ti, and Eu for the Eu-doped BCT ceramic (x = 0.025). The Au signal originates from the conductive coating applied prior to SEM/EDS analysis.

Figure 5 presents a representative EDS spectrum and elemental maps obtained for the BCT:Eu2.5 ceramic. The EDS spectrum confirms the presence of Ba, Ca, Ti and Eu. The Au signal originates from the gold coating applied to the sample prior to SEM/EDS examination. The elemental maps show a relatively uniform distribution of the major constituent elements throughout the analyzed area. Despite its low nominal concentration, the Eu signal is detectable across the mapped region. Due to the low Eu content and the limited accuracy of SEM-EDS for quantitative analysis of minor constituents, the EDS results are considered primarily qualitative.

The bulk density of the discussed ceramics, as well as relative density, which was calculated with respect to the theoretical density obtained from the refined lattice parameters, are listed in Table 3 and range from 93.7 to 97.1%.

The results presented in Table 3 indicate that Eu^3+^ doping does not significantly affect the densification of the material. For five of the six compositions, the relative density remains within a narrow range of 93.7–95.0%, while the sample with x = 0.020 exhibits a slightly higher value of 97.1%. Overall, the results suggest that Eu^3+^ incorporation does not lead to a systematic change in the densification behavior of BCT ceramics.

### 3.3. Impedance Spectroscopy Analysis

Micrographs of the microstructure of the ceramic materials under study showed that, with increasing Eu ion content, the grains become finer, which entails an increase in the fraction of grain boundaries. These changes are by no means without effect on the electrical properties of the ceramics studied. It is therefore of great importance to determine the contribution of grains and grain boundaries to the mechanisms responsible for electric charge transport [60,61]. For this purpose, complex impedance spectroscopy (CIS) was employed as a non-destructive characterization technique. Analysis of the electrical response over a wide frequency and temperature range enables the identification and separation of contributions from grains, grain boundaries, and electrode–material interfaces. This approach provides a more detailed understanding of the electrical conduction and relaxation processes occurring in the ceramics. The impedance response can be analyzed using four complementary formalisms: complex impedance (Z*), complex electric modulus (M*), complex admittance (Y*), and complex permittivity (ε*). These formalisms provide different representations of the same electrical response and facilitate the identification of individual contributions to the overall electrical behavior of the material.

**Figure 6:**
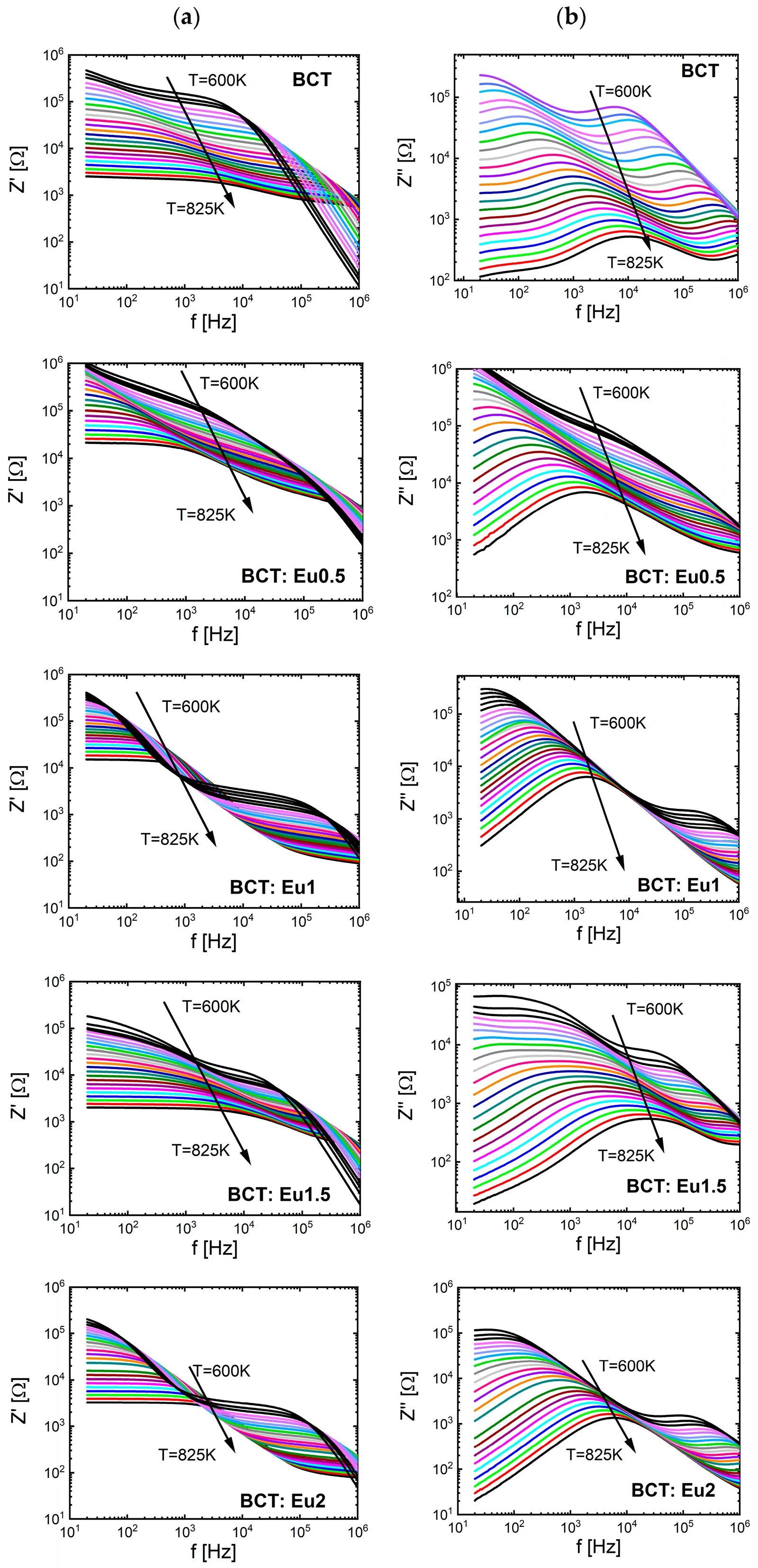

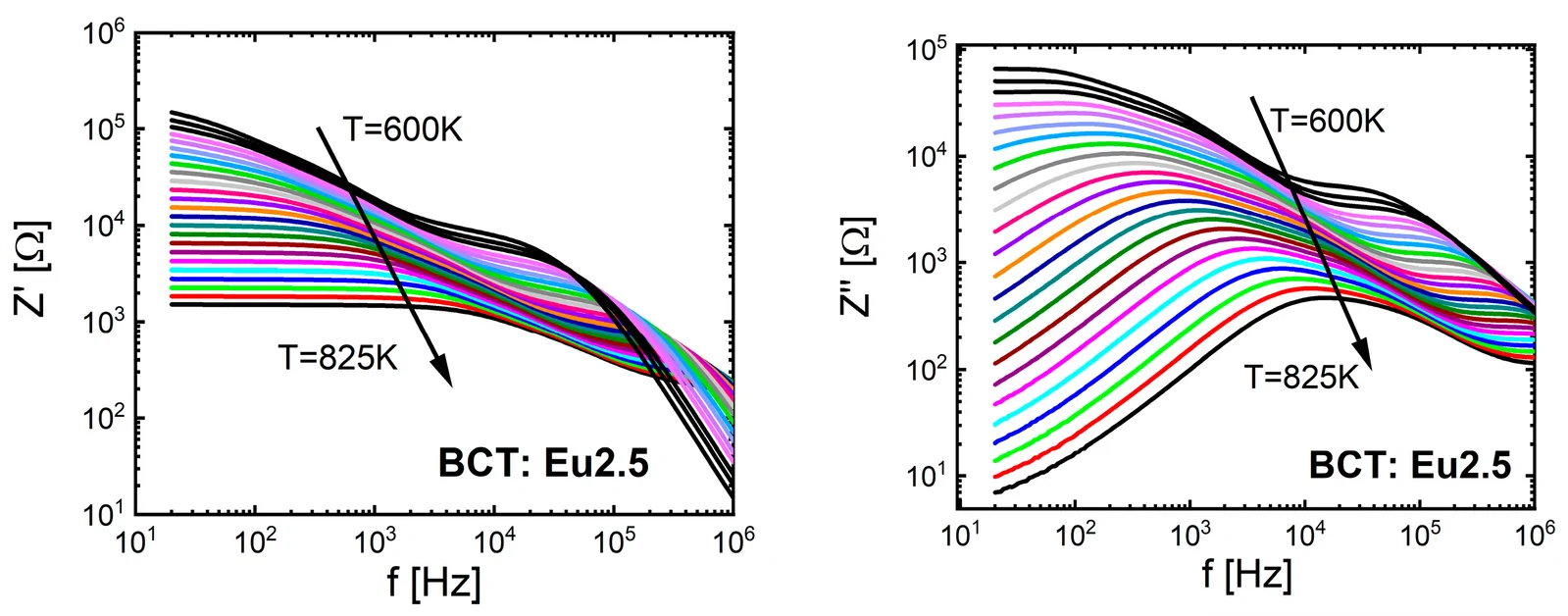
Frequency dependence of the real (Z′) (**a**) and imaginary (Z″) (**b**) impedance components of pure and Eu-doped (Ba_0.9−x_Eu_x_Ca_0.1_)TiO_3_ ceramics, measured for selected temperatures in the range of 600–825 K.

Figure 6 shows the frequency dependence of the real part and the imaginary part of the complex impedance of (Ba_0.9−x_Eu_x_Ca_0.1_)TiO_3_ ceramics with all investigated compositions, measured over the temperature range of 600–825 K. For all the investigated compositions, the value of Z′ decreases monotonically with increasing frequency, which is typical dispersive behavior for perovskite-structured ceramics. In the low-frequency range, Z′ exhibits a plateau region, the value of which systematically decreases with increasing temperature, indicating an increase in electrical conductivity with rising temperature—that is, a negative temperature coefficient of resistance (NTCR), characteristic of thermally activated electrical conduction in this class of materials [62]. It is also worth noting that the frequency range of the aforementioned plateau gradually broadens with increasing temperature. In the low-frequency region, the real part of impedance (Z′) exhibits a plateau that is independent of frequency, which is characteristic of a long-range DC conduction process [63,64]. In this frequency regime, charge carriers (primarily oxygen vacancies and thermally activated polarons) have sufficient time to complete long-range hops across energy barriers during a single cycle of the applied AC electric field. As the temperature increases from 600 K to 825 K, the magnitude of the Z′ plateau systematically decreases, demonstrating a negative temperature coefficient of resistance (NTCR) typical of thermally activated conduction in electroceramics [60,65].

Furthermore, the frequency range of the Z′ plateau gradually broadens toward higher frequencies with increasing temperature, reflecting an enhanced hopping rate and shorter relaxation times of mobile charge carriers [62,64]. Notably, the Z′ plateau is broadest in the undoped BCT ceramic (x = 0), indicating that the primary DC conduction mechanism is governed by intrinsic defect chemistry (i.e., oxygen vacancies formed during high-temperature sintering) rather than dopant-induced defects [66,67]. Upon Eu^3+^ substitution, the plateau narrows significantly, and dispersion occurs at lower frequencies. This shift is attributed both to the charge compensation mechanism via cation vacancies (V_Ba″_) [35,37] and to the pronounced grain refinement from 15.2 μm down to ∼1.4 μm, which greatly increases the grain-boundary density per unit volume and modifies the overall relaxation dynamics of the ceramic according to the brick-layer model [68,69].

The frequency dependences of the imaginary part of complex impedance (Z″) obtained for undoped (Ba_0.9_Ca_0.1_)TiO_3_ ceramics show two broadened maxima, which become sharper with increasing temperature (Figure 6b). The discussed maxima are connected with relaxation processes occurring in the material. The low-frequency maximum is ascribed to grain-boundary relaxation, whereas the higher-frequency peak corresponds to the grain contribution to the total relaxation [70]. The magnitude of both maxima decreases with the temperature, indicating the presence of space charge polarization at low frequency, which disappears at high frequency [71]. Moreover, the position of both maxima on the frequency axis (f_max_) depends on temperature; as the temperature rises, they shift towards the higher-frequency side, suggesting an increase in relaxation rate [72] and the thermal activation of this process [73]. This shift can be explained by the hopping conduction model in this material-the hopping rate of charge carriers increases with temperature, thereby significantly decreasing the relaxation time of mobile charges [74]. Similar behavior is observed for europium-modified ceramics; however, the second maximum, corresponding to grain relaxation, stays significantly more broadened. It is worth mentioning that for all discussed ceramics, at high temperatures, the peak corresponding to grain relaxation shifts beyond the upper limit of the measured frequency range, moving outside the high-frequency end of the spectra.

**Figure 7:**
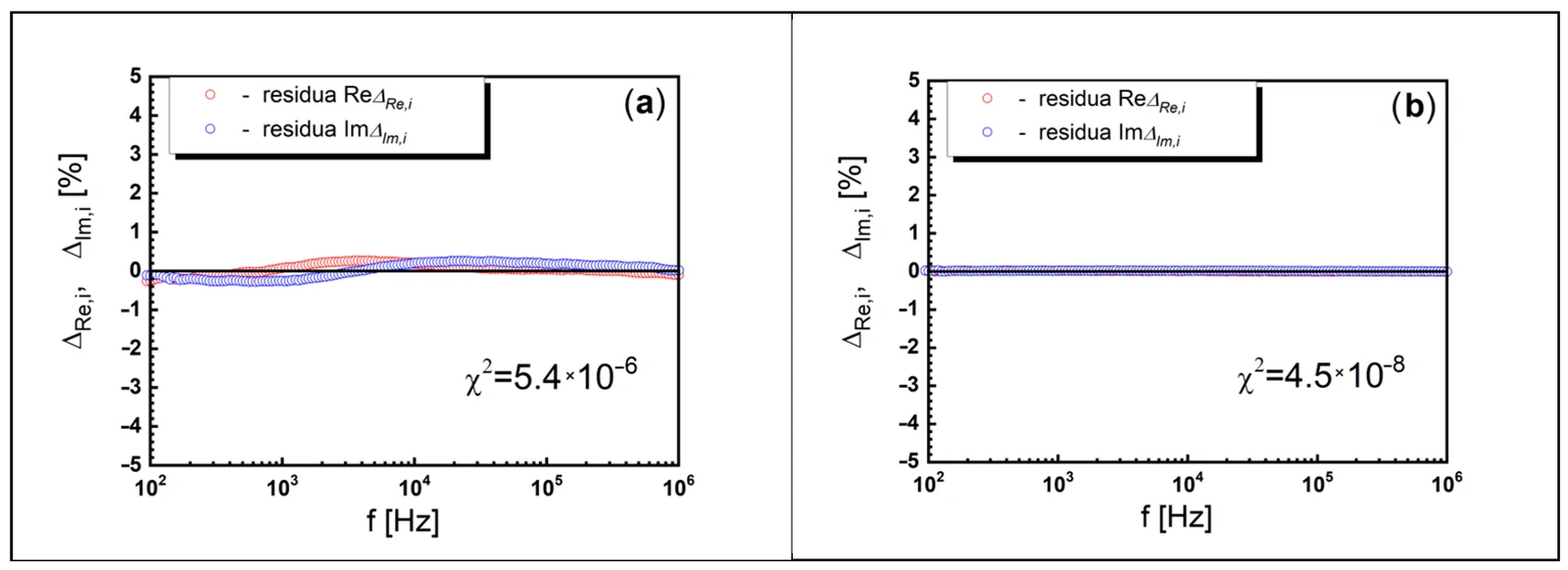
Frequency dependence of residuals of the real and imaginary parts of the impedance of (Ba_0.9−x_Eu_x_Ca_0.1_)TiO_3_ for x = 0 (**a**) and x = 0.025 (**b**) ceramics.

Impedance spectroscopy is well known for its high sensitivity to random noise, which often remains hidden within the raw impedance spectrum. Consequently, validating experimental data consistency is the next mandatory step for accurate impedance analysis [75,76]. In this study, the measurement quality was validated using the Kramers–Kronig (K-K) relations and the K-K test V1.01 program [77,78]. The recorded data demonstrated excellent agreement with the K-K transformations. The residual spectra calculated for the temperature of 650K, as an example, are shown in Figure 7. The test quality parameter was the coefficient χ^2^ [79].

**Figure 8:**
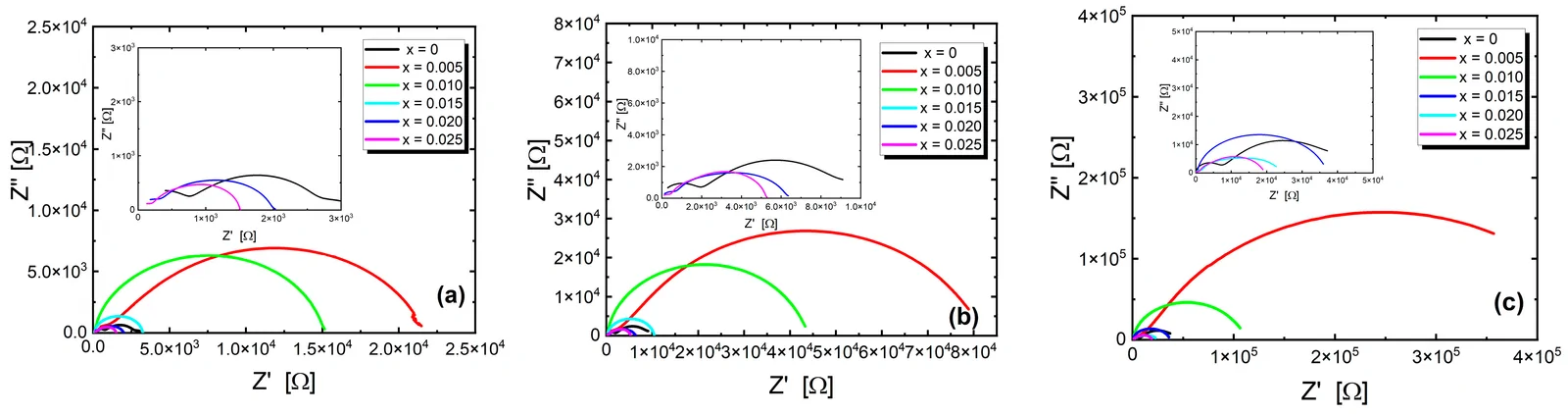
Nyquist plot for (Ba_0.9−x_Eu_x_Ca_0.1_)TiO_3_ ceramics, measured for selected temperatures: 800 K (**a**), 760 K (**b**), 700 K (**c**).

The aforementioned influence of grains and grain boundaries on the electrical properties of the discussed materials is much more clearly visible on the Nyquist plot (Figure 8). In the case of undoped ceramics, the plot consists of two deformed semicircles. The semicircle at the highest frequencies is associated with the electrical response of the grains; as the temperature increases, it progressively opens up, indicating that the upper frequency limit of the measuring apparatus is insufficient to capture the entire semicircle. The second semicircle, located in the lower frequency range and having a considerably larger radius, is associated with the grain boundaries [80].

With increasing europium ion content (starting from x = 0.01), the Nyquist plots exhibit a smaller semicircle, indicating a progressive decrease in the compound’s resistance. This decrease in resistance is accompanied by a corresponding reduction in the magnitude of both the real and imaginary impedance components. In addition, the relative size of the discussed semicircles changes: for an Eu^3+^ admixture equal to x = 0.005, the semicircle corresponding to grain-boundary relaxation progressively grows and becomes increasingly more deformed, while the semicircle associated with the grain relaxation undergoes a simultaneous decrease in size. For materials containing x = 0.01 and x = 0.015 Eu^3+^ ions, the previously mentioned semicircles merge into one strongly deformed semicircle, before splitting again, for higher dopant contents, into two semicircles associated with the grains and grain boundaries.

The shape of the presented Nyquist plots indicates that an equivalent electrical circuit composed of two Voigt elements connected in series should be used to describe the electrical properties of the discussed materials. These elements represent the contributions of grains and grain boundaries to the electrical properties [81].

**Figure 9:**
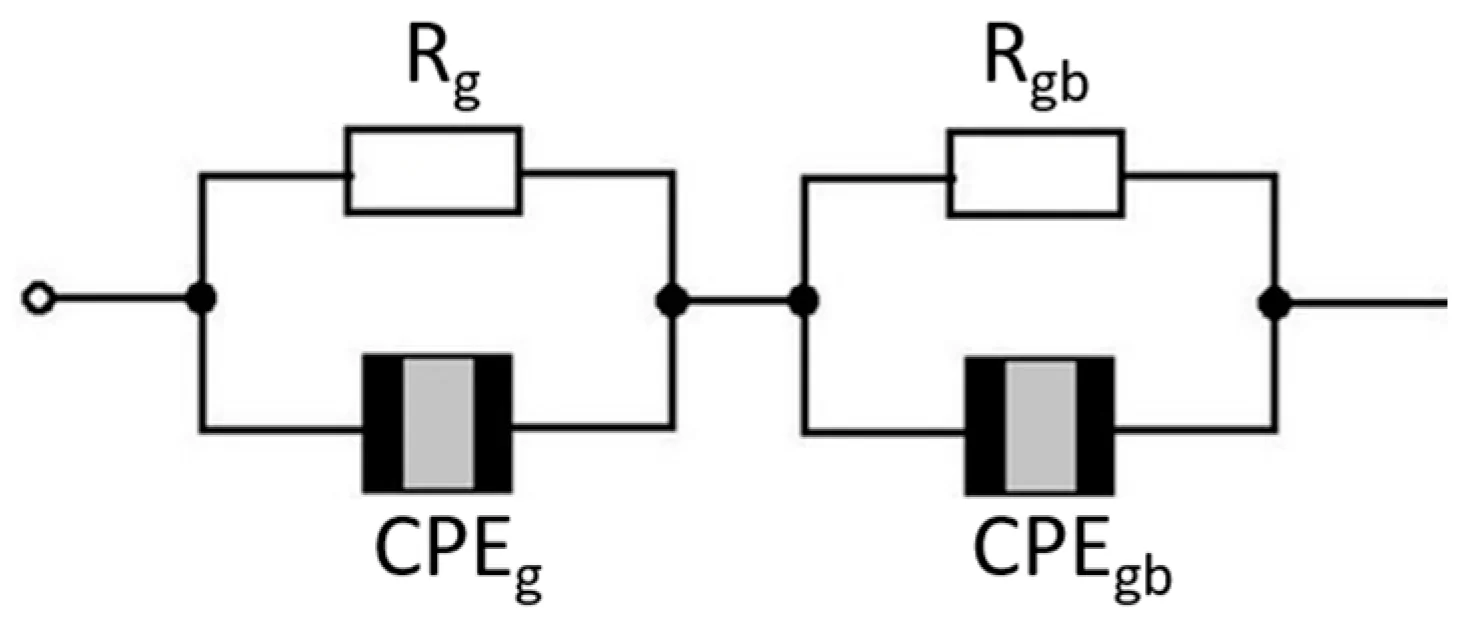
Electric equivalent system describing the electric properties of (Ba_0.9−x_Eu_x_Ca_0.1_)TiO_3_ ceramics.

**Figure 10:**
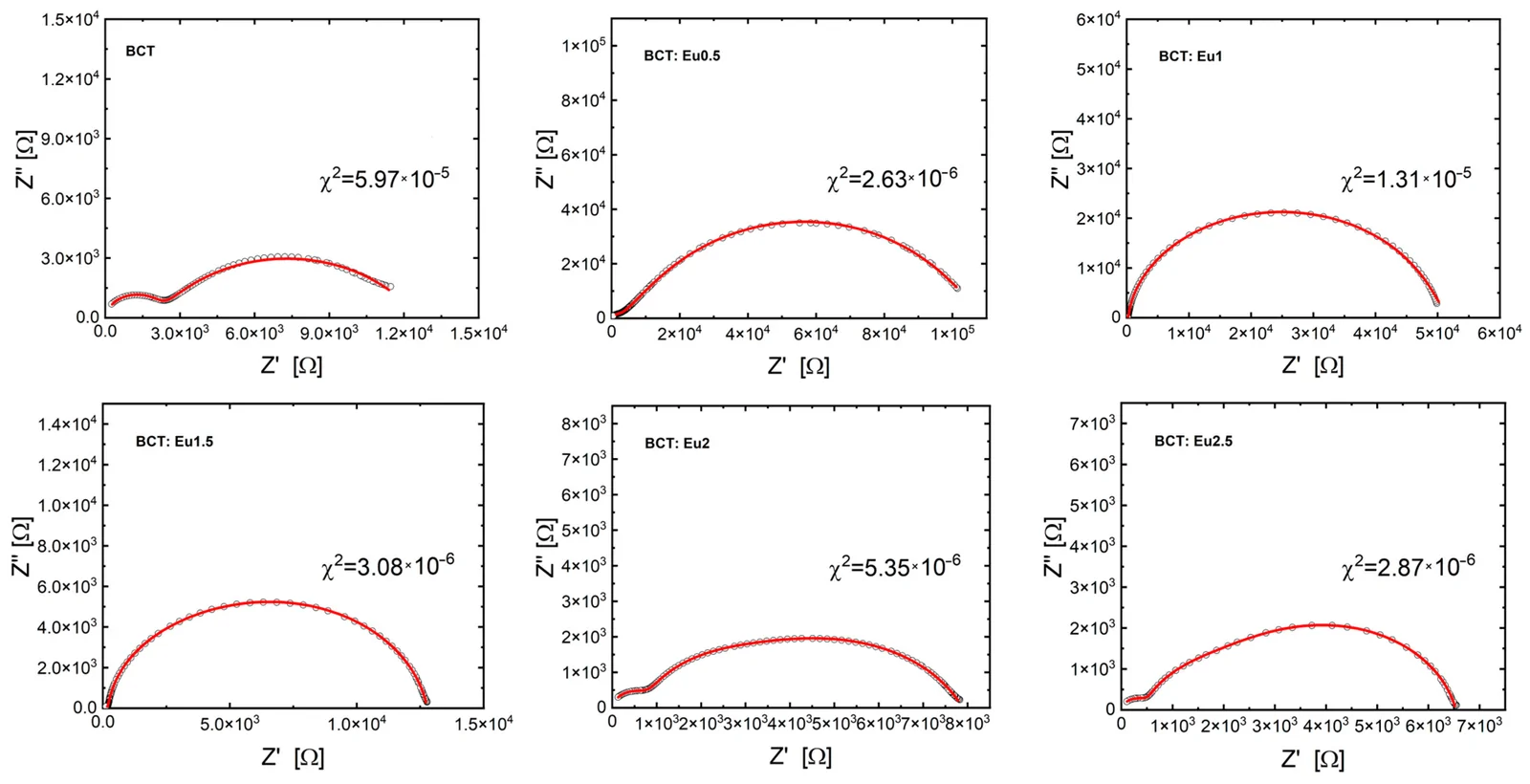
Experimental AC impedance spectrum in the complex plane (open circles) and modeled impedance spectrum using calculated values of the used equivalent circuit elements (solid red line) for (Ba_0.9−x_Eu_x_Ca_0.1_)TiO_3_ ceramics at 750 K.

The Z-View software (version 4.0g, Scribner Associates, Inc., Southern Pines, NC, USA) was used to obtain the parameters of each element of the equivalent circuit. High-quality fits were obtained only after replacing all capacitances with a constant phase element (CPE) (Figure 9). The impedance response of the CPE is defined as follows (4) [82]:

(4) $$Z_{\mathrm{CPE}}=\frac{1}{Q{\left(\mathrm{j\omega}\right)}^{n}}$$

where Q is the magnitude of the admittance (1/|Z|) at ω = 1 rad/s, and n is the CPE exponent (0 < n < 1); for *n* = 1 the element behaves as an ideal capacitor and Q = C. The symbol j denotes the imaginary unit, and ω the angular frequency [83,84]. Such non-ideal behavior of the microstructural elements, requiring description by means of a CPE, may result from the presence of more than one relaxation process [85,86]. Representative fits of the equivalent circuit to the experimental data obtained at 750 K for all investigated materials are presented in Figure 10. The figure also shows the corresponding values of the χ^2^ (chi-squared) parameter, which range from 2.63 × 10^−6^ to 5.97 × 10^−5^, indicating good agreement between the model and the measured data. Representative values of the fitted equivalent circuit parameters for all investigated samples at 700, 750 and 800 K are listed in Table S1 in the Supplementary Material. In every sample, R_gb_ exceeds R_g_, so the grain boundaries are the main barrier to charge carrier transport.

The introduction of Eu^3+^ ions at x = 0.005 causes a substantial increase in grain boundary resistance (from 37.8 kΩ to 471 kΩ at 700 K). This can be explained by the donor character of Eu^3+^ substituting for Ba^2+^/Ca^2+^ ions on the A sublattice. Compensation of the excess positive charge by cation vacancies, which segregate to the grain boundaries, leads to the formation of depletion layers (potential barriers) that hinder carrier transport [87]. In addition, donor doping lowers the concentration of oxygen vacancies, which are the main charge carriers in BCT at high temperatures [37,88,89]. For x = 0.01, the grain boundary resistance is still about three times higher than in undoped BCT (108 kΩ at 700 K). Starting from R_gb_ at x = 0.015, it shows a decreasing trend, and at x = 0.02 it falls below the value for undoped BCT. The decrease in R_gb_ at higher dopant concentrations may result either from partial incorporation of Eu^3+^ into the B sublattice, where it acts as an acceptor promoting the formation of oxygen vacancies [37,90], or from segregation of europium ions in the near-boundary regions, which lowers the potential barriers at the grain boundaries [91]. The second mechanism is supported by the reduction in grain size (segregating ions inhibit grain boundary migration) and by the increase in Q_gb_ for x ≥ 0.015, indicating a thinner depletion layer. A definitive conclusion, however, requires further investigation. The fitting errors for most parameters do not exceed 4%, and for the grain boundary parameters they are typically below 2%. The largest uncertainties concern R_g_ and Q_g_ in the samples with dopant contents of x = 0.01 and x = 0.015. This is to be expected in impedance spectra when the high-frequency (bulk) response is masked by the dominant grain boundary arc.

**Figure 11:**
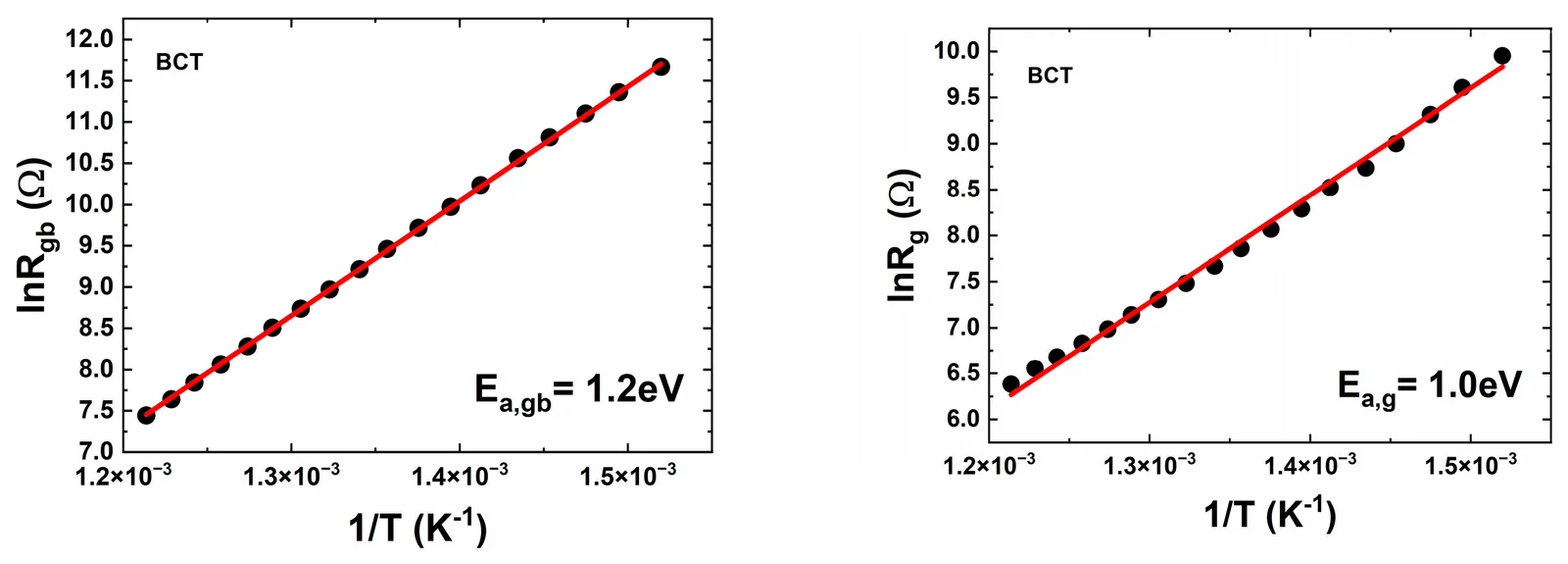

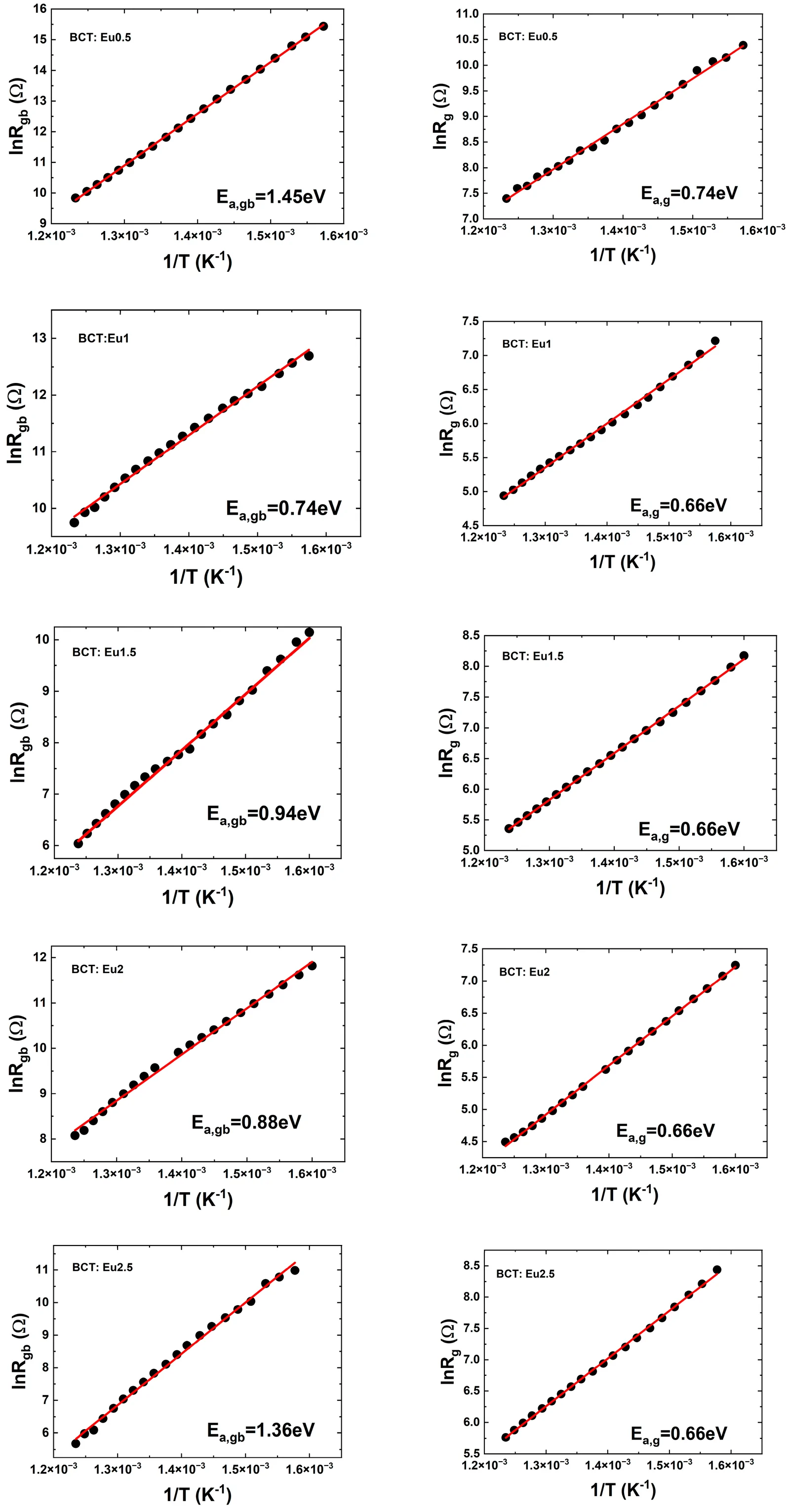
Dependence of the natural logarithm of the grain and grain-boundary resistances (R_g_ and R_gb_) on inverse temperature (1/T) for (Ba_0.9−x_Eu_x_Ca_0.1_)TiO_3_ ceramics, obtained from impedance spectra analysis.

In all studied materials, the grain resistance (R_g_) and grain boundary resistance (R_gb_) decrease monotonically with increasing temperature, which indicates the thermally activated nature of electrical conduction (Figure 10). This resistance–temperature dependence can be modeled using the Arrhenius relation (5):

(5) $$R=R_{0}\exp^{\left(\frac{E_{a}}{k_{B}T}\right)}$$

where E_a_ is the activation energy for conduction in grains or grain boundaries, k_B_ is Boltzmann’s constant, R_0_ is the pre-exponential factor and T is the temperature (K). Based on the Arrhenius formula, a linear fit was performed on the data presented in Figure 11, which made it possible to determine the values of the conduction activation energy for both the grains and the grain boundaries.

The activation energies of grain (E_a_,_g_) and grain-boundary (E_a,gb_) conduction determined from the Arrhenius plots (Figure 11) are summarized in Table 4, together with their difference Δ = E_a_,_gb_ − E_a,g_. For the undoped BCT ceramic, E_a,g_ ≈ 1.0 eV falls within the range commonly reported for the long-range migration of doubly ionized oxygen vacancies (V_O_^••^) in titanate perovskites [92,93,94]. This is consistent with the intrinsic defect chemistry of BaTiO_3_-based ceramics sintered at high temperature in air, which retain a small oxygen deficiency after cooling [53,66]. The grain-boundary activation energy is higher (E_a_,_gb_ ≈ 1.2 eV). Because the same charge carriers must pass through both regions, Δ ≈ 0.2 eV can be taken as an estimate of the additional potential barrier associated with the space-charge (double Schottky) layers at the grain boundaries [65,91,95]. In BaTiO_3_, the grain-boundary core carries an excess positive charge, so the positively charged oxygen vacancies are depleted in the adjacent space-charge layers. This accounts for the higher resistance and activation energy of the boundary regions [91].

The introduction of a small amount of europium (x = 0.005) sharply reduces activation energies of grain to 0.74 eV. Eu^3+^ substituting for Ba^2+^/Ca^2+^ acts as a donor (Eu_Ba_^•^). Under oxidizing conditions, its excess charge is compensated mainly by A-site vacancies (Equation (1)), and the Schottky-type equilibrium between cation and oxygen vacancies lowers the concentration of V_O_^••^ [35,67,88]. The decrease in activation energy therefore indicates a change in the dominant transport mechanism, from oxygen-vacancy migration towards a process with a lower energy barrier, most likely a partially electronic one involving electrons released from donor levels or Ti^3+^/Ti^4+^ small-polaron hopping [54,93,94]. Notably, the increase in E_a,gb_ is accompanied by a more than tenfold increase in R_gb_ (Table S1). The higher grain-boundary resistance at x = 0.005 therefore result from a higher energy barrier and not from a much larger pre-exponential factor R_0_ in Equation (5), i.e., a lower concentration of mobile carriers and/or a thicker resistive depletion layer, consistent with the accumulation of compensating cation vacancies near the boundaries [91,96]. Opposite changes in the activation energy and the pre-exponential factor are frequently observed in thermally activated transport and are described by the Meyer–Neldel compensation rule [97].

For x = 0.01, both activation energies reach their minimum values (E_a,g_ = 0.66 eV, E_a,gb_ = 0.74 eV), and their difference is small (Δ ≈ 0.08 eV). Because the two contributions have similar activation energies, their relaxation times (τ = RC) change with temperature in almost the same way, and their characteristic frequencies lie close together. This explains why the grain and grain-boundary semicircles merge into a single, strongly depressed arc in the Nyquist plots (Figure 8), and why R_g_ and Q_g_ carry the largest fitting uncertainties. The simultaneous minimum of both activation energies points to a common origin: at x = 0.01 the amount of Eu^3+^ incorporated into the A sublattice, and hence the donor-controlled modification of the defect structure, reaches its maximum. This composition may therefore indicate the approximate solubility limit of Eu^3+^ in the A-site sublattice of pure BCT.

Above this concentration, E_a,g_ remains constant at ≈ 0.66 eV, indicating that the defect chemistry of the grain interior no longer changes and that excess Eu^3+^ is not incorporated into the bulk. At the same time, E_a,gb_ increases again (0.94, 0.88 and 1.36 eV for x = 0.015, 0.02 and 0.025), and Δ grows to ≈ 0.7 eV for x = 0.025. This behavior indicates that excess europium accumulates at or near the grain boundaries, driven by the elastic size mismatch and by its electrostatic interaction with the boundary space charge [91,96]. The segregated dopant raises the effective barrier height and also exerts a solute-drag (pinning) effect on boundary migration during sintering. This agrees with the strongest grain refinement occurring in this concentration range (from 4.61 µm for x = 0.015 to 1.45 µm for x = 0.02, Figure 4). It is significant that E_a,gb_ for x = 0.025 (1.36 eV) exceeds the value of the undoped ceramic (1.2 eV), which was attributed to oxygen-vacancy migration. This suggests that oxygen vacancies again become the dominant carriers in the Eu-rich boundary regions. This can be rationalized by the amphoteric behavior of Eu^3+^, an ion of intermediate size [36,37,40]: at high local concentration, part of the europium may occupy the B site (Eu′_Ti_) and act as an acceptor compensated by V_O_^••^. Such a mechanism reconciles both explanations proposed above for the decrease in R_gb_ at high dopant contents. The grain-boundary resistance falls below that of undoped BCT despite the higher E_a,gb_ because the pre-exponential factor decreases, reflecting a higher carrier concentration in the boundary regions. The slight non-monotonicity at x = 0.02 (0.88 vs. 0.94 eV) may be related to the highest relative density (97.1%) and lowest closed porosity (0.8%) of this sample, which reduce the contribution of pores to the boundary response.

The proposed mechanism is consistent with previous reports on rare-earth doping of BaTiO_3_. Serna-Gallén et al. [90] showed by site-selective optical spectroscopy that Eu^3+^ preferentially occupies the A site of BaTiO_3_, with a solubility limit of about 3 mol%. The lower limit found here (x ≈ 0.01) may be related to the presence of Ca^2+^ on the A site, which reduces the average A-site radius and increases lattice strain, as well as to different processing conditions. A similar coincidence of a solubility limit and abrupt grain-growth suppression was reported by Nfissi et al. [98] for Ce-doped BaTi_0_._97_Y_0_._03_O_3_ ceramics.

## 4. Conclusions

The influence of Eu^3+^ doping on the structural, microstructural, and electrical properties of Ba_0.9_Ca_0.1_TiO_3_ ceramics (x = 0–0.025) was systematically investigated, with particular emphasis on the electrical transport and relaxation processes occurring within grains and grain boundaries. XRD analysis confirmed the predominantly tetragonal perovskite structure (P4mm) of the investigated ceramics. Increasing Eu content resulted in a gradual reduction in tetragonal distortion, accompanied by pronounced grain refinement. The average grain size decreased from 15.2 μm for undoped BCT to approximately 1.4 μm at the highest Eu concentrations, indicating a significant influence of europium on microstructural development. Impedance spectroscopy revealed that the electrical response of all investigated ceramics is governed by thermally activated conduction, exhibiting a negative temperature coefficient of resistance (NTCR). Two relaxation processes associated with grains and grain boundaries were identified. The experimental impedance spectra were successfully described using an equivalent circuit consisting of two Voigt elements connected in series, incorporating constant phase elements to account for non-ideal relaxation behavior. For all compositions, grain-boundary resistance exceeded grain resistance, demonstrating that grain boundaries constitute the principal barriers to charge carrier transport. The effect of Eu doping on grain-boundary resistance was strongly concentration-dependent. At x = 0.005, the grain-boundary resistance increased from 37.8 kΩ for undoped BCT to 471 kΩ at 700 K. Further Eu addition progressively reduced this resistance, which eventually fell below that of the undoped ceramic at x = 0.02. These results demonstrate that even small amounts of europium substantially modify the electrical response of grain boundaries. Arrhenius analysis revealed a pronounced decrease in the activation energies of grain conduction following Eu incorporation. The minimum values of 0.66 eV and 0.74 eV, respectively, were obtained at x = 0.01, compared with 1.00 eV and 1.20 eV for undoped BCT. At higher Eu concentrations, the grain activation energy remained approximately constant, whereas the grain-boundary activation energy increased again, reaching 1.36 eV at x = 0.025. This divergence indicates that further Eu addition affects electrical transport in the grain interiors and grain-boundary regions differently. The simultaneous minimum in activation energies at x = 0.01, followed by their different concentration dependences, suggests a change in the dominant electrical transport processes. These observations may be associated with modifications in defect chemistry, charge compensation, and the electrical characteristics of grain boundaries. Possible changes in Eu site occupancy or dopant distribution may also contribute to this behavior; however, the present results do not provide direct evidence for a specific solubility limit or Eu segregation at grain boundaries. Overall, the results demonstrate that Eu^3+^ doping provides an effective approach to modifying the microstructure and electrical transport properties of BCT10 ceramics. In particular, the distinct responses of grains and grain boundaries highlight the importance of dopant concentration in controlling the electrical behavior of these materials.

## Figures and Tables

**Table 1:** Ionic radii of the ions considered in this study [48].

Ion	Site in the Perovskite	Ionic Radius [Å]
Ba^2+^	A	1.61
Ca^2+^	A	1.34
Ti^4+^	B	0.605
Eu^3+^	B (hypothetical)	0.947
Eu^3+^	A (hypothetical)	1.12

**Table 2:** Structural parameters derived from Rietveld refinement of XRD data for Eu-doped BCT samples. a, b, c—lattice constants; V—the volume of the unit cell; Δ—tetragonal deformation; R_e_, R_p_, R_wp_—agreement indices of the refinement procedure.

Sample	Space Group	a = b [Å]	c [Å]	V [Å^3^]	Δ [%]	R_e_, R_p_, R_wp_ [%]
BCT	P4mm	3.9792	4.0241	63.72	1.13	3.2, 7.8, 9.3
BCT:Eu0.5	P4mm	3.9784	4.0238	63.69	1.14	3.3, 7.9, 9.6
BCT:Eu1	P4mm	3.9788	4.0228	63.68	1.11	3.4, 6.8, 8.4
BCT:Eu1.5	P4mm	3.9787	4.0195	63.63	1.03	3.5, 7.3, 9.6
BCT:Eu2	P4mm	3.9797	4.0161	63.61	0.91	3.7, 7.4, 9.2
BCT:Eu2.5	P4mm	3.9795	4.0114	63.53	0.80	4.0, 6.9, 8.8

**Table 3:** Theoretical density (ρ_th_), bulk density determined by the Archimedes method (ρ_A_), relative density determined by the Archimedes (ρ_rel_), open (P_open_, measured), total (P_tot_ = 100 − ρ_rel_), and closed (P_closed_ = P_tot_ − P_open_) porosity.

x (Eu)	ρ_th_ [g/cm^3^]	ρ_A_ [g/cm^3^]	ρ_rel_ [%]	P_open_ [%]	P_tot_ [%]	P_closed_ [%]
0	5.824	5.481	94.1	4.3	5.9	1.6
0.005	5.828	5.526	94.8	4.2	5.2	1.0
0.010	5.831	5.541	95.0	2.3	5.0	2.7
0.015	5.837	5.481	93.9	2.1	6.1	4.0
0.020	5.841	5.669	97.1	2.1	2.9	0.8
0.025	5.850	5.482	93.7	2.4	6.3	3.9

**Table 4:** Activation energies of grain (E_g_) and grain-boundary (E_gb_) conduction for (Ba_0.9−x_Eu_x_Ca_0.1_)TiO_3_ ceramics and their difference Δ = E_gb_ − E_g_.

x (Eu)	E_g_ [eV]	E_gb_ [eV]	Δ = E_gb_ − E_g_ [eV]
0	1.00	1.20	0.20
0.005	0.74	1.45	0.71
0.010	0.66	0.74	0.08
0.015	0.66	0.94	0.28
0.020	0.66	0.88	0.22
0.025	0.66	1.36	0.70

## Data Availability

The original contributions presented in this study are included in the article/Supplementary Material. Further inquiries can be directed to the corresponding author.

## Supplementary Materials

The following supporting information can be downloaded at: https://www.mdpi.com/article/10.3390/ma19204394/s1, Table S1: BCT–BCT:Eu2.5
