# Supplementary material for: Influence of Europium Doping on the Electrical Properties of Ba0.9Ca0.1TiO3 Ceramics
Source: Materials (Basel). 2026 Oct 19;19(20):4394. doi: 10.3390/ma19204394 (PMC13706903; doi:10.3390/ma19204394)
Supplement: Supplementary file 1 [file materials-19-04394-s001.zip › materials-4585516-Table S1.pdf]

Undoped BCT

Fitting parameters - rounded values

T [K]	Parameter	Value	Error [%]
**800**	$R_g\ [\Omega]$	606	1,9
	$Q_g [S \cdot s^n]$	$3{,}37 \cdot 10^{-10}$	4,8
	n_g	0,9285	0,33
	$R_{gb}\ [\Omega]$	1861	0,21
	$Q_{gb}\ [S \cdot s^n]$	$3{,}24 \cdot 10^{-7}$	1,6
	n_{gb}	0,6732	0,23
**750**	$R_g\ [\Omega]$	2106	0,42
	$Q_g\ [S \cdot s^n]$	$2{,}08 \cdot 10^{-10}$	3,1
	n_g	0,9644	0,21
	$R_{gb}\ [\Omega]$	10336	0,38
	$Q_{gb}\ [S \cdot s^n]$	$2{,}05 \cdot 10^{-7}$	2,2
	n_{gb}	0,6646	0,34
**700**	$R_g\ [\Omega]$	6487	0,67
	$Q_g\ [S \cdot s^n]$	$1{,}52 \cdot 10^{-10}$	3,5
	n_g	0,9908	0,26
	$R_{gb}\ [\Omega]$	$37{,}83 \cdot 10^{3}$	0,84
	$Q_{gb}\ [S \cdot s^n]$	$4{,}72 \cdot 10^{-8}$	3,5
	n_{gb}	0,6722	0,60

BCT:Eu0.5

Fitting parameters - rounded values

T [K]	Parameter	Value	Error [%]
**800**	R_g [Ω]	839	2,4
	Q_g[$S{\cdot}s^n$]	$1{,}05{\cdot}10^{-9}$	8,3
	n_g	0,867	1,8
	R_{gb} [Ω]	$23{,}14{\cdot}10^{3}$	0,69
	Q_{gb} [$S{\cdot}s^n$]	$3{,}32{\cdot}10^{-8}$	0,65
	n_{gb}	0,7865	0,20
**750**	R_g [Ω]	$2{,}07{\cdot}10^{3}$	5,6
	Q_g [$S{\cdot}s^n$]	$1{,}02{\cdot}10^{-9}$	9,4
	n_g	0,865	1,3
	R_{gb} [Ω]	$101{\cdot}10^{3}$	0,27
	Q_{gb} [$S{\cdot}s^n$]	$3{,}17{\cdot}10^{-8}$	0,96
	n_{gb}	0,7673	0,20
**700**	R_g [Ω]	$4{,}54{\cdot}10^{3}$	4,0
	Q_g [$S{\cdot}s^n$]	$8{,}64\ {\cdot}10^{-10}$	3,2
	n_g	0,878	1,4
	R_{gb} [Ω]	$471{\cdot}10^{3}$	0,53
	Q_{gb} [$S{\cdot}s^n$]	$2{,}84{\cdot}10^{-8}$	1,5
	n_{gb}	0,7505	0,30

BCT:Eu1

Fitting parameters - rounded values

T [K]	Parameter	Value	Error [%]
**800**	R_g [Ω]	139,6	2,0
	Q_g[S·s^n]	$2{,}42\cdot10^{-9}$	9,4
	n_g	0,648	2,5
	R_{gb} [Ω]	17136	0,072
	Q_{gb} [S·s^n]	$3{,}43\cdot10^{-8}$	3,1
	n_{gb}	0,7296	0,60
**750**	R_g [Ω]	273,1	0,79
	Q_g [S·s^n]	$3{,}37\cdot10^{-7}$	9,5
	n_g	0,6124	0,98
	R_{gb} [Ω]	50576	0,17
	Q_{gb} [S·s^n]	$2{,}14\cdot10^{-8}$	4,1
	n_{gb}	0,7493	0,87
**700**	R_g [Ω]	463,1	0,86
	Q_g [S·s^n]	$1{,}52\cdot10^{-9}$	9,3
	n_g	0,7480	0,77
	R_{gb} [Ω]	$(108\cdot10^{3}$	0,32
	Q_{gb} [S·s^n]	$1{,}20\cdot10^{-8}$	2,1
	n_{gb}	0,873	1,2

BCT:Eu1.5

Fitting parameters - rounded values

T [K]	Parameter	Value	Error [%]
**800**	R_g [Ω]	419,3	2,0
	Q_g[$S{\cdot}s^n$]	$2{,}41{\cdot}10^{-8}$	4,6
	n_g	0,8949	0,38
	R_{gb} [Ω]	879,7	0,87
	Q_{gb} [$S{\cdot}s^n$]	$4{,}79{\cdot}10^{-8}$	0,72
	n_{gb}	0,9008	0,16
**750**	R_g [Ω]	1530	1,1
	Q_g [$S{\cdot}s^n$]	$3{,}48{\cdot}10^{-8}$	1,9
	n_g	0,8588	0,22
	R_{gb} [Ω]	4534	0,38
	Q_{gb} [$S{\cdot}s^n$]	$6{,}81{\cdot}10^{-8}$	0,61
	n_{gb}	0,8708	0,14
**700**	R_g [Ω]	$2{,}93{\cdot}10^{3}$	4,1
	Q_g [$S{\cdot}s^n$]	$3{,}71{\cdot}10^{-8}$	5,0
	n_g	0,8706	0,71
	R_{gb} [Ω]	$15{,}59{\cdot}10^{3}$	0,89
	Q_{gb} [$S{\cdot}s^n$]	$1{,}29{\cdot}10^{-7}$	1,6
	n_{gb}	0,7771	0,46

BCT:Eu2

Fitting parameters - rounded values

T [K]	Parameter	Value	Error [%]
**800**	$R_g\ [\Omega]$	450	3,4
	$Q_g [S{\cdot}s^n]$	$2,85{\cdot}10^{-8}$	1,6
	n_g	0,9988	0,83
	$R_{gb}\ [\Omega]$	2719	1,9
	$Q_{gb}\ [S{\cdot}s^n]$	$2,69{\cdot}10^{-8}$	2,2
	n_{gb}	0,91758	0,11
**750**	$R_g\ [\Omega]$	719	3,1
	$Q_g\ [S{\cdot}s^n]$	$1,53{\cdot}10^{-8}$	3,7
	n_g	0,9961	0,90
	$R_{gb}\ [\Omega]$	11883	0,37
	$Q_{gb}\ [S{\cdot}s^n]$	$2,79{\cdot}10^{-8}$	0,82
	n_{gb}	0,89815	0,046
**700**	$R_g\ [\Omega]$	$8,60{\cdot}10^{3}$	2,9
	$Q_g\ [S{\cdot}s^n]$	$1,74{\cdot}10^{-8}$	2,9
	n_g	0,9929	0,59
	$R_{gb}\ [\Omega]$	$27,86{\cdot}10^{3}$	1,5
	$Q_{gb}\ [S{\cdot}s^n]$	$6,58\ {\cdot}10^{-8}$	2,7
	n_{gb}	0,8555	0,16

BCT:Eu2.5

Fitting parameters - rounded values

T [K]	Parameter	Value	Error [%]
**800**	R_g [Ω]	294	3,3
	$Q_g [S \cdot s^n]$	$9,1 \cdot 10^{-9}$	11
	n_g	0,990	1,6
	R_{gb} [Ω]	1762	1,7
	$Q_{gb} [S \cdot s^n]$	$1,24 \cdot 10^{-7}$	1,5
	n_{gb}	0,7448	0,34
**750**	R_g [Ω]	1922	2,7
	$Q_g [S \cdot s^n]$	$2,49 \cdot 10^{-8}$	3,4
	n_g	0,8646	0,47
	R_{gb} [Ω]	5256	1,1
	$Q_{gb} [S \cdot s^n]$	$2,46 \cdot 10^{-7}$	0,98
	n_{gb}	0,7183	0,36
**700**	R_g [Ω]	$8,01 \cdot 10^{3}$	4,3
	$Q_g [S \cdot s^n]$	$4,14 \cdot 10^{-8}$	4,8
	n_g	0,7956	0,79
	R_{gb} [Ω]	$14,73 \cdot 10^{3}$	2,8
	$Q_{gb} [S \cdot s^n]$	$4,53 \cdot 10^{-7}$	3,5
	n_{gb}	0,694	1,5
